# Supplementary material for: A case-controlled study investigating gender differences in Face Name Hobby Recall (FNHR) performance in healthy community-dwelling older adults
Source: PLOS Ment Health. 2025 Mar 18;2(3):e0000244. doi: 10.1371/journal.pmen.0000244 (PMC12798586; doi:10.1371/journal.pmen.0000244)
Supplement: Attachment — Submitted filename: Response to Reviewers.docx [file pmen.0000244.s001.docx]

Dear PLOS Mental Health editorial team, reviewer #1, and Dr. Sandra Paola Mondragón Bohórquez,

Thank you for your consideration of our work, your time, and your valuable comments and suggestions. Below we have responded to all of the points raised by each of the reviewers. We made every effort to comprehensively address their concerns and revise our submission accordingly.

We would like to start by saying that all questions/comments of the reviewers are shown in italics below, all page and line numbers referenced in this document refer to the page and line numbers in clean version of the revised manuscript, and final figures have been run through the Preflight Analysis and Conversion Engine (PACE) digital diagnostic tool.

**General Comments**

**Question/Comment 1:** *Explicitly link your findings and outcomes to well-being. A direct link to mental health and well bring is one of our publishing criteria (*[*https://journals.plos.org/mentalhealth/s/criteria-for-publication#loc-1*](https://journals.plos.org/mentalhealth/s/criteria-for-publication#loc-1)*). This will need to be addressed to be considered in-scope for PLOS Mental Health.*

As suggested, we have more explicitly linked our findings and outcomes to well-being to align with PLOS Mental Health publishing criteria. On page 29, lines 431-439 we stress that associative memory, particularly face-name memory, is an important cognitive function for navigating the world and a strong predictor of neurodegenerative diseases that further harm mental health and overall well-being. Our study sheds more light on associative memory in older adults, a surprisingly understudied topic considering that this age group is extremely susceptible to mental health and neurodegenerative disorders. We also reiterate the connection between associative memory and depression.

**Comments from Reviewer #1**

**Question/Comment 1:** *Line 48: “involvement of select neuroanatomical structures and brain networks [3,4,5].” It would be beneficial for the reader to get some examples of involved brain structures and networks.*

We added specifics including the following text “including the fusiform face area, superior temporal sulcus, and the anterior temporal face patch” at the end of the indicated sentence (now found on page 3, lines 50-51) and added citation 2 to the existing in-text citations for that line. This article, A Neural Basis of Facial Action Recognition in Humans by Srinivasan, Golomb, and Martinez, provides evidence for the function of the superior temporal sulcus in facial recognition, while the other citations support the roles of the fusiform face area and anterior temporal face patch.

**Question/Comment 2:** *Lines 49-52 introduce the reader to the different age groups (children, young-, middle-aged, and older adults). However, the age range of each group is unknown to the reader, making it difficult to interpret previous and current findings. The authors are encouraged to further describe the age groups by age range.*

The age ranges of these age groups were not strictly defined in the published meta-analysis from which we summarized past findings. The published meta-analysis compiled data from more than sixty studies of face-name associative memory and the authors of these studies had different samples and different definitions themselves. We reworked this introductory paragraph to make the research landscape with respect to children, young-, middle-aged, and older adults more clear and included approximate age ranges based on the compiled data. To see the extensive revisions addressing this comment see page 3, lines 52-66.

**Question/Comment 3:** *Line 76: “with ethical standards” is written twice. Please correct.*

The indicated typographical error was corrected.

**Question/Comment 4:** *The paragraph “Study participants” does not describe the participants involved in this study but rather participants involved in the main study “Web-LABrainS”. Authors are encouraged to find a different title for this paragraph*

We agree that this section title could lead to some confusion. This paragraph has been renamed “Web-LABrainS participants and study subset” and a sentence was added before the final sentence to more clearly indicate that the participants in the current study were selected from the larger study and underwent a case-controlled matching process on page 5, lines 103-105.

**Question/Comment 5:** *In the paragraph “Face Name Hobby Recall (FNHR) test”, it is unclear if the memory task was self-administered or administered by lab personnel. More details are necessary.*

We apologize for the confusion over the language surrounding the manner in which the FNHR test was conducted. We hope the following changes have clarified this matter. In the first sentence of this section, we state that the test is self-guided and cite a previous feasibility study of the larger web-LABrainS study from which we selected the subjects whose data was included in the current study. This study touches on the validation of the FNHR test and how it is conducted. In addition to this, we have revised the final line of the first paragraph of this section (page 6, lines 130-131) to state “In total, subjects completed three trials of the FNHR assessment in each session, all of which were self-administered over the course of a single session using the remote, unsupervised web-LABrainS cognitive assessment tool.” Web-LABrainS and its cognitive assessment tool are also described in the previous section as “web-based” and “self-guided”.

**Question/Comment 6:** *Lines 103-105: “The order of images in terms of race and gender was as follows: Asian female, white male, biracial female, white female, white male, white male, black male, and black female.” Why were participants shown faces of four different females in terms of race (i.e., Asian female, biracial female, White female, Black female), but only two different male faces (i.e., White male, Black male)? In order to look at memory performance of sex-specific face differences, male and female faces should be equally distributed.*

When designing the web-based Louisiana Aging Brains Study, and the cognitive and health assessments it contains, the overall goal was to have numerous short assessments that could be done quickly to get a global picture of cognitive function and health of the individual. The primary purpose of including the FNHR test was to quickly and objectively assess associative memory. Efforts were made to equally distribute the faces by gender and to include faces of multiple races. However, when designing the instrument, we did not anticipate its use for performing an in-depth assessment of gender and racial biases in face recognition. In the current study we made interesting findings about gender bias, and because the instrument was balanced for gender, we included these findings among our results. In our current limitations section, we do recognize that the FNHR assessment isn't balanced across races and would likely benefit in the future (in a subset of participants) to include a larger number of items to adequately represent faces of different ages, races, genders and their combinations on page 32, lines 505-511.

**Question/Comment 7 and 8:** *In the paragraph “Measurement of depressive symptoms”, it is unclear which instrument was used to measure depressive symptoms. Authors should add the specific instrument and its reference for scoring to the paragraph.*

*Tables 1 and 2 show characteristics of the study sample. However, various scores are mentioned that have not been described in the methods section (i.e., Comfort with Computers, Mobility, Driving Frequency, Prescription Medications Taken, Medication Label Literacy, Quality of Life, Memory Concern), making it hard for the reader to interpret them. All characteristics listed in tables 1 and 2 should also be described in the methods section in order for the reader to be able to interpret the results.*

The web-LABrainS depressive symptom assessment is a novel assessment adapted for use in an online, self-guided format. It was psychometrically validated against the Geriatric Depression Scale and the Geriatric Anxiety Inventory in our past feasibility study, which we have now cited on page 7, lines 149-150. This section has been adjusted to explain this and to more thoroughly explain the scoring of the assessment. We have now also renamed the “Measurement of depressive symptoms” section to “Other web-LABrainS measures” to include details on all characteristics listed in Tables 1 and 2 as requested. Tables 1 and 2 were also reformatted to include more information about the scoring scales, and Table 1 was additionally reformatted to include p-values referenced in Figure 1. We hope these changes address concerns about the readers’ ability to interpret all of the measures included.

**Question/Comment 9:** *Figures 1 and 2 show correlation results. However, the methods do not include correlations in the analysis section. All analytical approaches should be mentioned in the methods section.*

We mistakenly included “correlation” in the title of Figure 1. This has now been corrected. The statistical methodology for the correlation findings in Figure 2 (lines 203-206) and explanations of the analyses performed for Figures 1 (lines 197-202), 4 and 5 (lines 229-237) and Table 1 (lines 195-197) have now been included in the “Statistical analyses” section.

**Question/Comment 10 and 11:** *Lines 317-319: “The three faces for which male and female subjects had the highest level of correct name identification were a male face and two female faces (Rob, Amy, and Cindy) (Fig 5).” The three names do not need to be listed here since the reader does not know the full list of names used in this paradigm.*

*Figure 5 lists eight names. If those are the names used in the memory paradigm in this study, they should be included in the method section and described (e.g., common name in the study area, etc.).*

We agree that including the full list of names and hobbies in the “Face Name Hobby Recall (FNHR) test” section of the Materials and Methods section is beneficial. We included these lists starting on page 5, line 112. Because this list is now included earlier in the paper as well as in Figure 5, it now seems appropriate to continue listing the names corresponding to the three faces in the Results section.

**Question/Comment 12:** *The discussion section does not provide explanations/suggestions 1) why OA females were better in detecting faces compared to OA males, and 2) why OA males better detected female vs. male faces compared to younger male participants. The discussion would benefit from some possible interpretations and suggestions.*

Further interpretations and suggestions were provided in the discussion section on pages 29-30, lines 446-451 and page 30, lines 457-465. Face-name associative memory is understudied in older adults, and the literature that does exist on this topic is mixed. Foremost, we suggest more research in this area. Our results are not necessarily inconsistent with the literature on older adults, but our gender bias findings diverge from findings in other age groups. More research is needed to understand the differences between older adults and younger adults for whom there is much more scientific literature, but we do tentatively provide an explanation related to past studies of how emotion recognition changes with age.

**Comments from Dr. Sandra Paola Mondragón Bohórquez (Reviewer #2)**

**Question/Comment 1:** *Describe significant physical examination and important clinical findings.*

The web-LABrainS assessment is part of an online, self-guided longitudinal study. It contains demographic questions, survey items, a psychometrically validated depressive symptom assessment, and other psychometrically validated cognitive assessments like the FNHR test (the focus of the current paper). To reiterate, web-LABrainS has no physical exams or in-person clinical evaluations as the sole purpose is to provide an alternative to such in-clinic visits. Reviewer #1 also raised concerns about the clarity surrounding how these assessments are conducted. We addressed these concerns by added more language about how web-LABrainS as a whole and specific assessments like the FNHR test are “web-based”, “self-guided”, and “unsupervised” (page 6, lines 130-131). We also cite our feasibility study in which all of the relevant assessments are psychometrically validated against existing pen-and-paper/face-to-face assessments.

**Question/Comment 2:** *In this study, historical and current information from this episode of care was described and organised as a time line.*

We were not entirely sure how to interpret this comment. We thought it was best for the reader if we described the order of assessments and the return sessions in the longitudinal study in chronological order. Reviewer #1 also raised some concerns about readers potentially being confused about differentiating the overall longitudinal study (web-LABrainS) from the current study and focus of this article. To address this, we renamed the “Study participants” section to “Web-LABrainS participants and study subset” and added a sentence before the final sentence to more clearly indicate that the participants in the current study were selected from the larger study and underwent a case-controlled matching process (page 5, lines 103-105).

**Question/Comment 3:** *In order to perform the two-factor variance analysis, it is important to explain the values of the assumptions of homogeneity and normal distribution within the groups.*

The use of mixed-model ANOVA with 2x3 factorial design in our study is a preliminary analysis. The implementation of this test in our code has a Greenhouse-Geisser correction for sphericity, which we have now specified on page 9, lines 213-214. Our sample size is sufficiently large enough that the central limit theorem can reasonably account for issues with non-normality. Additionally, because of our rigorous matching process, we have identical sample sizes in each of our groups. Equal group sizes help combat violations of homoscedasticity because they reduce the influence of heteroscedasticity (unequal variances across groups) on statistical analyses by balancing the contribution of each group to the overall variance estimation [1,2]. Substantial research has also been done to show that ANOVA is robust in the absence of these parametric assumptions, especially with equal and large sample sizes [3,4].

We follow up this analysis with post-hoc paired t-tests at each of our time points to determine whether there are significant differences in FNHR scores between the paired male and female subjects at those time points. The resulting p-values undergo further Bonferroni correction for multiple comparisons. These results are what we report, not the results of the ANOVA itself.

We believe that our methods are appropriate for our data and are better than alternatives.

1. Sawyer SF. Analysis of Variance: The Fundamental Concepts. *Journal of Manual & Manipulative Therapy*. 2009;17(2):27E-38E. doi:[10.1179/jmt.2009.17.2.27E](https://doi.org/10.1179/jmt.2009.17.2.27E)

2. BOX GEP. NON-NORMALITY AND TESTS ON VARIANCES. *Biometrika*. 1953;40(3-4):318-335. doi:[10.1093/biomet/40.3-4.318](https://doi.org/10.1093/biomet/40.3-4.318)

3. Arnold SF. Asymptotic Validity of F Tests for the Ordinary Linear Model and the Multiple Correlation Model. *Journal of the American Statistical Association*. Published online December 1, 1980. Accessed December 13, 2024. <https://www.tandfonline.com/doi/abs/10.1080/01621459.1980.10477568>

4. Akritas MG, Papadatos N. Heteroscedastic One-Way ANOVA and Lack-of-Fit Tests. *Journal of the American Statistical Association*. 2004;99(466):368-382. doi:[10.1198/016214504000000412](https://doi.org/10.1198/016214504000000412)

**Authorship Addition**

Due to his substantial contributions, Dr. Robbie Beyl has been removed from the Acknowledgements section and added to the authorship list. His information is now included on the title page.

**Conclusions**

We would like to thank you all again for taking the time to review our work and consider it for publication. We believe we have comprehensively addressed your concerns and look forward to the final editorial decision in the near future.
